# An unusual cause of renal colic: ovarian teratoma

**DOI:** 10.1590/S1677-5538.IBJU.2018.0141

**Published:** 2018

**Authors:** Pablo Garrido-Abad, Miguel Ángel Rodríguez-Cabello, Arturo Platas Sancho, Mairena Coronado Ruiz, Juan José Ortiz Zapata

**Affiliations:** 1 Department of Urology, Hospital Sanitas La Moraleja, Madrid, Spain; 2 Department of Gynecology. Hospital Sanitas La Moraleja, Madrid, Spain; 3 LABCO (SYNLAB) Pathology. Madrid, Spain

## CASE PRESENTATION

**Figure 1:**
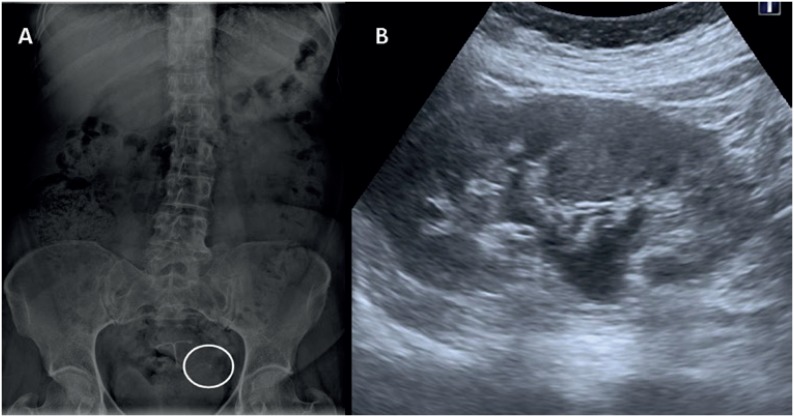
A) Abdominal X-ray with left pelvic calcification (white circle); B) Ultrasound image showing grade II left ureterohydronephrosis.

**Figure 2:**
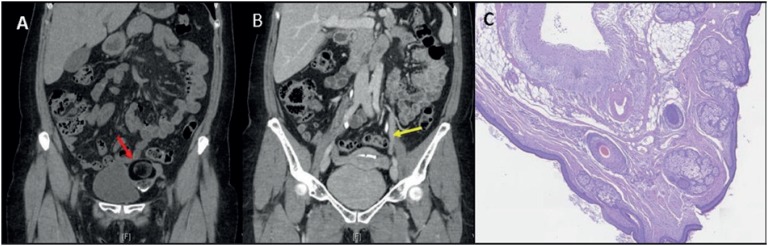
A) Coronal abdominopelvic CT scan image revealing 5 cm left ovarian MCT (red arrow); B) dilated left gonadal vein (yellow arrow); C) Histology of ovarian McT: mature hair follicles, sebaceous glands, fat cells and mature nervous tissue.

A 56-year-old female presented with a complaint of left flank pain for two weeks. Her past medical history was unremarkable. A left pelvic calcification was observed on abdominal X-ray (Figure-1A). Ultrasonographic examination (Figure-1B) revealed a grade II left ureterohydronephrosis and heterogeneous cystic mass in left ovary, and CT was performed to confirm diagnosis, showing a well-defined 5.4 × 4.3 × 4.5 cm left adnexal lesion (Figure-2A) with fat and calcification, compressing distal ureter and gonadal vein that were dilated (Figure-2B) as a consequence of the compression by ovarian mass. Tumor markers (CA125, CEA and CA19-9) were with in normal range. Under a clinical diagnosis of ovarian germ cell tumor, laparoscopic salpingo-ooforectomy was performed. Histopathological examination of the specimen (Figure-2C) revealed mature hair follicles, sebaceous glands, fat cells and mature nervous tissue, typical features of a mature cystic teratoma (MCT).

Ovarian MCT is a cystic or solid tumor (composed of mature, adult type tissues) which accounts for 10-20% of all ovarian tumors (1). Malignant transformation occurs in less than 2% (2). Ovaries are close to pelvic urological organs, such as ureter and bladder, so ovarian masses can often impinge upon these adjacent organs and develop symptoms like pain, urinary and gastrointestinal complaints (1). Ovarian cancer is described as the most common cause of malignant extrinsic ureteral obstruction (16.6%) (3), but the exact prevalence of ureteral involvement by ovarian MCT is still unknown. The differential diagnosis of calcifications in abdominal plain films of the female pelvis include: vascular calcifications (atherosclerosis, calcified aneurysms, phleboliths), those originating from the urinary tract (ureterolithiasis and vesical lithiasis), inflammatory masses (epiploic calcifications, dropped gallstones, foreign bodies) and nodal calcifications (4).

Early diagnosis and treatment in terms of a conservative surgical approach is recommended. Ovarian MCT should be considered in the differential diagnosis of distal ureteric obstruction causing proximal hydroureteronephrosis in young female patients (5).
